# Cancer kill code extension

**DOI:** 10.1016/j.omtn.2023.08.003

**Published:** 2023-08-20

**Authors:** Marcus E. Peter

**Affiliations:** 1 Department of Medicine/Division Hematology/Oncology, Department of Biochemistry and Molecular Genetics, Feinberg School of Medicine, Northwestern University, Chicago, IL, USA

Short interfering RNAs (siRNAs) silence targeted genes through RNA interference (RNAi). Beyond eliciting cytotoxicity via the activation of an interferon response,^1^ siRNAs can also induce general toxicity in cells when they contain specific sequence motifs (i.e., UGGC).^2^ While such siRNAs kill cells through RNAi in a sequence-specific fashion, the exact mechanism of their toxicity is unclear.

Recently, a universal kill code was identified based on a 6-mer seed usually found in miRNAs (position 2–7 of the guide strand). An extensive analysis of all 4,096 possible combinations of 6-mer seeds within a short double-stranded RNA scaffold tested in arrayed screens of human and murine cancer cell lines was performed.^3^^,^^4^ G-rich seeds stood out as the most potent sequences in killing cancer cells by targeting C-rich seed matches enriched in the 3' UTR of critical survival genes through a process called death induced by survival gene elimination (DISE).^5^ Many of these toxic 6-mer seeds were found to be part of canonical 7-mer seeds present in tumor-suppressive miRNAs.^3^^,^^4^ Corbin et al. validated the "kill code" for prostate cancer cells. They observed that these toxic seeds were specifically active against survival genes induced by androgen signaling.^6^ Interestingly, this form of DISE, referred to as androgen network DISE, was selectively effective against prostate cancer cells, leaving normal prostate epithelial cells unaffected. This finding aligns with the observation that DISE-inducing short RNAs, when administered to mice via nanoparticles, do not cause adverse effects on normal tissues.^7^ Corbin et al. have now expanded the screen of miRNA seeds to 7-mers by performing a pooled screen of 15,572 shRNAs containing most of the 16,384 possible combinations of 7-nt seeds, identifying the ones most effective at inhibiting cell growth of prostate cancer cells.^8^

Upon validating some of these RNAi active sequences, it was revealed that the toxic effect is associated with seed sequence complementarity to the 3′ UTR of both essential and androgen receptor (AR) coregulatory genes. The authors analyzed two of these 7-mer seed sequences in more detail: GUGUGUA and UGUUUGC. They found that introducing siRNAs containing either of these two seeds into prostate cancer cells *in vitro*, or the UGUUUGC seed *in vivo* in xenografted mice, led to more effective growth inhibition than knocking down AR or individual AR coregulatory genes. These data suggest the potential use of these newly identified toxic short RNAs as a therapeutic strategy for treating prostate cancer.

While this new screen has significantly increased the number of possible sequences that could be used to treat prostate cancer, the rationale behind the existence of these specific sequences is still unclear. In contrast to the toxicity of 6-mer seeds, which likely arose from the coevolution of miRNAs and the nucleotide composition of 3′ UTRs, drawing a link between 7-mer seed-containing short RNAs and an evolutionarily conserved mechanism is less straightforward. However, the discovery of these sequences, which as short RNAs target both androgen signaling regulatory and essential gene networks, may pave the way for developing new RNAi-based therapeutics to treat advanced prostate cancer that has become resistant to castration and chemotherapy.
